# Mortality in adolescents after therapeutic intervention for self-harm: A systematic review and meta-analysis

**DOI:** 10.1002/jcv2.12302

**Published:** 2025-01-23

**Authors:** Faraz Mughal, Paul Young, Daniel Stahl, Joan R. Asarnow, Dennis Ougrin

**Affiliations:** 1 School of Medicine, Keele University, Keele, UK; 2 NIHR Greater Manchester Patient Safety Research Collaboration (PSRC), University of Manchester, Manchester, UK; 3 Centre for Mental Health and Safety, Division of Psychology and Mental Health, University of Manchester, Manchester, UK; 4 School of Sport, Exercise, and Health Sciences, Loughborough University, Loughborough, UK; 5 Department of Biostatistics and Health Informatics, Institute of Psychiatry, Psychology, and Neuroscience, Kings College London, London, UK; 6 Department of Psychiatry and Biobehavioural Sciences, University of California, Los Angeles, California, USA; 7 Youth Resilience Unit, Queen Mary University of London, London, UK

**Keywords:** adolescents, mortality, self-harm, suicide attempts, therapeutic intervention

## Abstract

**Background:**

Self-harm in adolescents is an international concern. Evidence highlights that therapeutic intervention (TI), such as cognitive behaviour therapy informed treatments, after self-harm leads to reduced self-harm repetition. However, there is no prior literature about the effects of TI on future mortality in adolescents. We examined the effect of TI on mortality rates in adolescents across RCTs.

**Methods:**

This review was reported in accordance with PRISMA guidance. MEDLINE, EMBASE, PsycINFO, and Cochrane Library were searched to 19 June 2024. Two authors independently screened titles, abstracts, and full texts against predefined criteria. RCTs were included if they compared a TI versus a comparator in adolescents up to 18 years with at least one prior self-harm episode. There was no lower age limit. For the pooled effect size of mortality, the DerSimonian-Laird method was used, and a random effects model for self-harm and suicide attempts. The primary outcome was intra or post-trial mortality in adolescent post TI, and the effect of TIs on self-harm including attempted suicide episodes were secondary outcomes. Analyses were done in Stata.

**Results:**

Twenty-four trials of TIs consisting of 3470 randomised adolescents were included. The pooled risk difference for mortality of participants in the TI group was 0.002 (95% CI −0.003 to 0.008, *p* = 0.42). There were 6 deaths in the TI group compared to 15 deaths in the comparator group. The pooled risk difference for TI on repeat self-harm was −0.07 (95% CI −0.132 to −0.007, *p* = 0.028), and −0.05 (95% CI −0.086 to −0.007, *p* = 0.022) for suicide attempts compared to comparator.

**Conclusions:**

This review found no significant impact of TIs on future mortality in adolescents. We also demonstrated that TIs can reduce suicide attempts which can lead to substantial benefits for adolescents, families, and clinical services.

## Introduction

Self-harm, defined as self-injury or poisoning irrespective of motive, is an international priority in adolescents (Knipe et al., 2022; National Institute For Health and Care Excellence, 2004). Previous self-harm (including suicide attempts and non-suicidal self-injury) is strongly associated with death by suicide: increasing the likelihood up to 50 times compared to the general population (Hawton et al., 2015). Suicide occurs throughout the lifespan, but has a greater proportional influence on mortality within younger age ranges (Rodway et al., 2016). Suicide is the second leading cause of death among 15-29-year-olds and the third leading cause of death in 15–19-year-olds globally (World Health Organization, 2014).

A recent national study indicated that more than half of young people who die by suicide have a history of self-harm (National Confidential Inquiry into Suicide and Homicide by People with Mental Illness (NCISH), 2017). Adolescents who have previously self-harmed are nine times more likely to die from unnatural causes, and 34 times more likely to die from fatal poisoning (Morgan et al., 2017). Some randomised controlled trials (RCT) of TIs have reported reduced mortality in adolescents who received an TI at long term follow-up (King et al., 2019). The effect, however, of TIs for self-harm on future mortality in adolescents has not been rigorously studied.

A 2015 meta-analysis of therapeutic interventions (TI) (defined as replicable pharmacological or psychosocial interventions such as cognitive behaviour therapy (CBT) informed treatments) for self-harm in adolescents found that TI after self-harm led to reduced repetition of self-harm when compared to treatment as usual (Ougrin et al., 2015). There have been a number of TIs evaluated for adolescents after self-harm, with mixed results, since this review was conducted (searches completed May 2014), and so it remains useful to understand how TIs influence self-harm repetition (Cottrell et al., 2020; Santamarina-Perez et al., 2020).

We aimed in this meta-analysis to compare follow-up mortality rates among adolescents who received a TI for self-harm with those who had not across RCTs. In addition, we examined the effect of TIs on repeat self-harm episodes in adolescents. We hypothesised there would be lower mortality rates among adolescents who received a TI compared to those who did not. These findings can provide new and updated evidence about the effectiveness of TIs for adolescents after self-harm and inform future intervention strategies.

## Methods

### Protocol

The protocol was registered with PROSPERO (CRD42020178764). This review was conducted and reported in accordance with Preferred Reporting Items for Systematic Reviews and Meta-Analyses (PRISMA) guidance (Moher et al., 2009).

### Data sources and search strategy

Searches were conducted in four electronic databases: MEDLINE, EMBASE, PsycINFO, and Cochrane Library from inception to initially 14 May 2020 using refined and tailored search strategies for each database. Searches were updated on 19 June 2024.

The following subject headings or MeSH keywords were incorporated into searches: *Self-injurious behaviour*; *Self-harm*; *Self-mutilation*; *Suicide*; *Suicidal ideation*; *Suicide attempted*; *Drug overdose*; *Poisoning*; *Adolescent*; *Child*; *Therapeutics*; *RCT*. The search strategies are listed in Appendix S1. The search term ‘self-harm’ encompassed the terms suicide attempts, non-suicidal self-injury, and self-harm with undetermined intent; adhering to the National Institute for Health and Care Excellence definition of self-harm: self-poisoning or injury, irrespective of the apparent purpose of the act (National Institute For Health and Care Excellence, 2004).

No language or location restrictions were applied. To ensure that all relevant studies were identified, reference lists of eligible studies were hand-searched. Additional RCTs not captured in the searches but known to co-authors DO and JA (topic experts) were also screened for inclusion.

### Study screening and selection

Studies were included if they met the following criteria: RCTs comparing a TI defined as a replicable psychosocial or pharmacological intervention versus comparator condition (Ougrin et al., 2015); in adolescents up to 18 years that had engaged with treatment following at least one episode of self-harm. Trial papers with the longest follow-up data published were included over the original trial publication.

Studies were excluded if they were pilot studies; less than 50% of the participant sample had engaged in self-harm prior to inclusion in the study; and studies in which most of the participants had neurological or developmental disorders, for example, autism.

**Figure 1:**
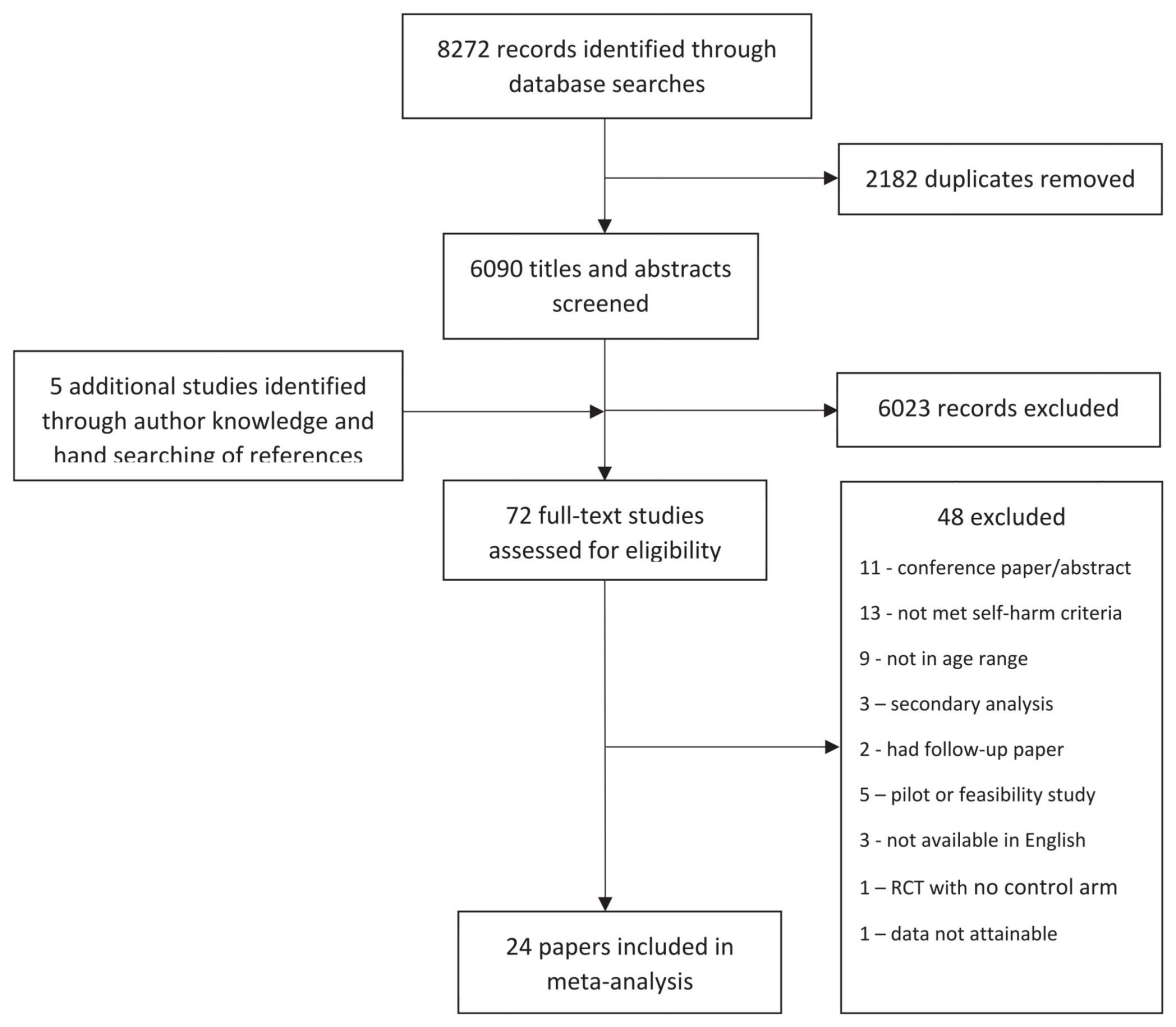
PRISMA flow chart.

Two authors (FM and PY) independently reviewed all titles and abstracts, and then full texts, against predefined eligibility criteria in a two-staged approach. Discrepancies were resolved through discussion with a third author (DO). Corresponding authors were contacted directly for data clarification queries at full text screening stage. Reasons for excluded studies after full text review are stated in the PRISMA flow chart (Figure 1). Study screening was managed in Endnote X9 (The Endnote Team, 2013).

### Data extraction and assessment of bias

Eligible full-text studies were subjected to data extraction and assessment of bias independently by two authors (FM and PY) on a pre-piloted Excel spreadsheet. Data were extracted on study setting, country, and aim; participant characteristics; type of intervention and delivery; intervention components; number of participants randomised to trial arms; adherence to intervention; repeat self-harm and suicide attempt individual participant and mean episodes; and mortality in participants at longest follow-up. For most published, studies mortality data was not reported in the paper and therefore FM, PY, and DO emailed study authors to request data. We defined mortality as death by any cause, including suicide.

Assessment of bias was undertaken using the Cochrane Collaboration’s tool for assessing the risk of bias: https://handbook-5-1.cochrane.org/chapter_8/table_8_5_a_the_cochrane_collaborations_tool_for_assessing.htm which captures domains of selection, performance, detection, attrition, and reporting bias. Discrepancies across study domains were resolved through discussion. To assess the influence of bias on outcomes we pooled the six items into a total score by summing up the number of high or unknown bias which can range from 0 to 6.

### Outcomes

The primary outcome was intra-or-post trial mortality among included participants. The study hypothesis was generated prior to the commencement of this meta-analysis. The effect of TIs on repeat self-harm and suicide attempts were examined as post-hoc secondary outcomes.

### Statistical analysis

As an absolute effect size, we calculated the risk difference which is the difference of observed risks (proportions of individuals with the outcome of interest) in the two groups. The risk difference is asymptotically normally distributed and does not require a transformation in meta-analyses. Due to the small number of observed mortalities, the DerSimonian-Laird method was used because restricted maximum likelihood (REML) estimation did not converge (DerSimonian & Laird, 1986). Absolute mortality numbers in individual RCTs were concealed to protect patient confidentiality. (Office for National Statistics) Pooled overall effect sizes for self-harm and suicide attempts were estimated by a random-effects model using REML estimation (Borenstein et al., 2021). Analyses for self-harm and suicide attempts were kept separate because of how they were reported in included studies.

A random-effect analysis model assumes that individual studies are estimating different treatment effects due to the diversity of clinical interventions and methodological factors. We calculated the *I*^2^ statistic to estimate total variation across studies that is due to heterogeneity relative to pure sampling variation (Higgins & Thompson, 2002). *I*^2^ describes the percentage of total variation across studies that is due to heterogeneity rather than sampling error and ranges between 0% (no inconsistency) and 100% (high heterogeneity) with values of 25%, 50%, and 75% suggesting low, moderate, and high heterogeneity.

Results are presented as forest plots which shows study-specific risk differences and the overall effect size with their respective confidence intervals, information about study heterogeneity, and the significance of the overall test.

Finally, meta-regression was performed to assess the influence of group therapy (only group vs. not only group) and TIs with family treatment components (present or not present) on the risk difference of self-harm (Higgins et al., 2019).

### Sensitivity analyses

Sensitivity analyses were conducted by repeating the meta-analyses omitting one study at a time to investigate the influence of a single study on the overall effect size estimate. We also assessed the influence of trial bias by assessing the influence of the bias score on the risk difference for self-harm.

### Publication and other bias

Statistically significant results are more likely to be published than studies with non-significant results. Therefore, the presence of publication bias was assessed informally by visual inspections of funnel plots, which represent a plot of effect size on the *x*-axis against a study’s precision (standard error) on the *y*-axis. The absence of studies in the right bottom corner (low precision and small treatment effect sizes (here positive risk differences)) of a funnel plot are usually taken as an indication of publication bias.

We also used the Duval and Tweedie non-parametric ‘trim and fill’ method of accounting for potential publication bias in a meta-analysis by estimating the number of missing studies, imputing values for missing studies and estimating a revised effect size (Duval & Tweedie, 2000). If the conclusion of the meta-analysis remains unchanged following adjustment for the publication bias using the trim and fill method, the results can be considered robust, excluding major publication bias.

Data were analysed using Stata using the meta command (Statacorp, 2021).

## Results

### Search results and study characteristics

The searches yielded 6090 unique citations post deduplication, of which 72 full texts were assessed for eligibility, and 24 studies were included for meta-analyses (Figure 1 for PRISMA flow chart). (Asarnow et al., 2011, 2017; Bjureberg et al., 2023; Chanen et al., 2008; Cottrell et al., 2020; Diamond et al., 2010, 2019; Esposito-Smythers et al., 2011, 2019; Goldstein et al., 2024; Green et al., 2011; Hazell et al., 2009; Kennard et al., 2018; King et al., 2006, 2019; McCauley et al., 2018; Mehlum et al., 2019; Ougrin et al., 2013; Pineda & Dadds, 2013; Rockstroh et al., 2023; Rossouw & Fonagy, 2012; Santamarina-Perez et al., 2020; Stallard et al., 2024; Wood et al., 2001) The characteristics of included studies are listed in Table 1. The RCTs included 3470 randomised participants, of which 1729 were randomised to a TI. RCTs were conducted in USA (Asarnow et al., 2011, 2017; Diamond et al., 2010, 2019; Esposito-Smythers et al., 2011, 2019; Goldstein et al., 2024; Kennard et al., 2018; King et al., 2006, 2019; McCauley et al., 2018) (*N* = 11), UK (Cottrell et al., 2020; Green et al., 2011; Ougrin et al., 2013; Rossouw & Fonagy, 2012; Stallard et al., 2024; Wood et al., 2001) (*N* = 6), Australia (Chanen et al., 2008; Hazell et al., 2009; Pineda & Dadds, 2013) (*N* = 3), Norway (Mehlum et al., 2019) (*N* = 1), Spain (Santamarina-Perez et al., 2020) (*N* = 1), Sweden (Bjureberg et al., 2023) (*N* = 1), and Germany (Rockstroh et al., 2023) (*N* = 1).

Most included RCTs (*n* = 21) compared TIs to treatment as usual or enhanced usual care except three where active comparators were used: manualised good clinical care (Chanen et al., 2008); individual and group supported therapy (McCauley et al., 2018); and family-enhanced nondirective supportive therapy (Diamond et al., 2019). The included RCTs tested a variety of TIs: family-focused (Asarnow et al., 2011; Asarnow et al., 2017; Cottrell et al., 2020; Diamond et al., 2010, 2019; Pineda & Dadds, 2013) (*N* = 6), group based (Green et al., 2011; Hazell et al., 2009; Wood et al., 2001) (*N* = 3), youth nominated support team (King et al., 2006, 2019) (*N* = 2), cognitive analytical therapy (Chanen et al., 2008) (*N* = 1), CBT (Bjureberg et al., 2023; Esposito-Smythers et al., 2011, 2019) (*N* = 2), DBT (McCauley et al., 2018) (*N* = 1), DBT-A (Goldstein et al., 2024; Mehlum et al., 2019; Santamarina-Perez et al., 2020) (*N* = 3), mentalisation-based therapy for adolescents (Rossouw & Fonagy, 2012) (*N* = 1), programmes informed by both CBT and DBT (Rockstroh et al., 2023; Stallard et al., 2024) (*N* = 2), the ‘as safe as possible’ intervention focussed on emotion regulation and safety planning with post-discharge mobile phone app support (Kennard et al., 2018) (*N* = 1), and therapeutic assessment (Ougrin et al., 2013) (*N* = 1). Two TIs were single session interventions (Kennard et al., 2018; Ougrin et al., 2013). 16 RCTs included a family treatment component within interventions (Asarnow et al., 2011, 2017; Bjureberg et al., 2023; Cottrell et al., 2020; Diamond et al., 2010, 2019; Esposito-Smythers et al., 2011, 2019; Kennard et al., 2018; King et al., 2006, 2019; McCauley et al., 2018; Mehlum et al., 2019; Pineda & Dadds, 2013; Rossouw & Fonagy, 2012; Santamarina-Perez et al., 2020).

17 RCTs provided sufficient data for meta-analyses about self-harm; six on suicide attempts; and 24 about mortality. Across all RCTs, four scored low (0) on bias across all six domains, none in one, two in two, six in three, six in four, and six in five. Table 2 lists the risk of bias scores for each RCT.

### Mortality meta-analysis

**Figure 2:**
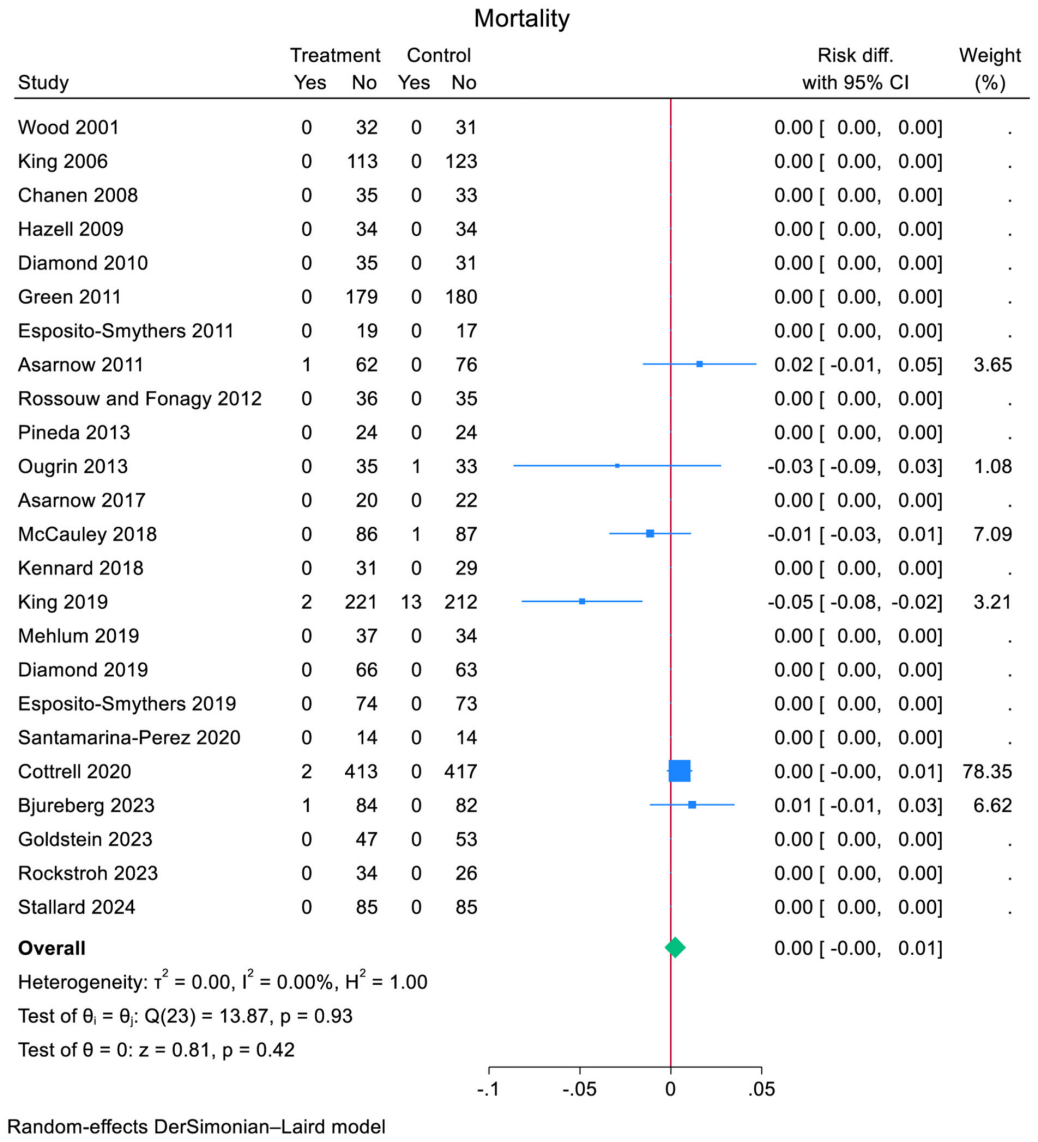
Effect of TI for self-harm versus comparator condition on mortality in adolescents.

Data on mortality was obtained from all RCTs (*N* = 24). Mortalities were observed in six (25%) RCTs. In the TI group, 6 (0.33%) out of 1822 participants died while in the comparator group 15 (0.82%) out of 1829 participants died. The pooled risk difference for mortality of participants in the TI group was 0.0024 (95% CI, −0.0035 to 0.0083, *z* = 0.81 *p* = 0.42, using DerSimonian–Laird estimator, *I*^2^ = 0) (see Figure 2). All RCTs provided data for mortality and in 18 trials there were no observed events. RCTs with no mortality events contributed no information about the risk ratio. Further analyses were not feasible due to the small number of events.

### Repeat self-harm meta-analyses

**Figure 3:**
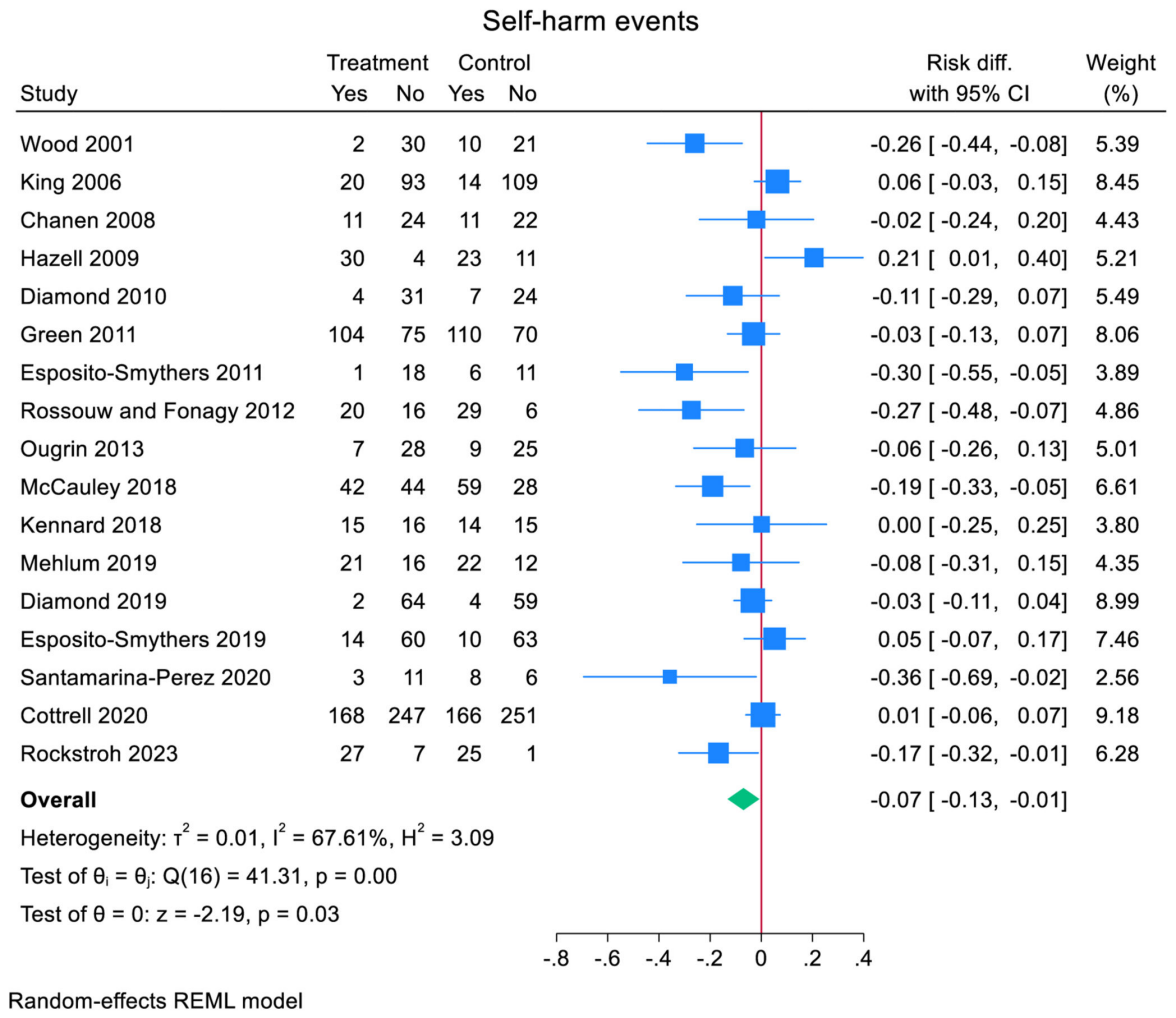

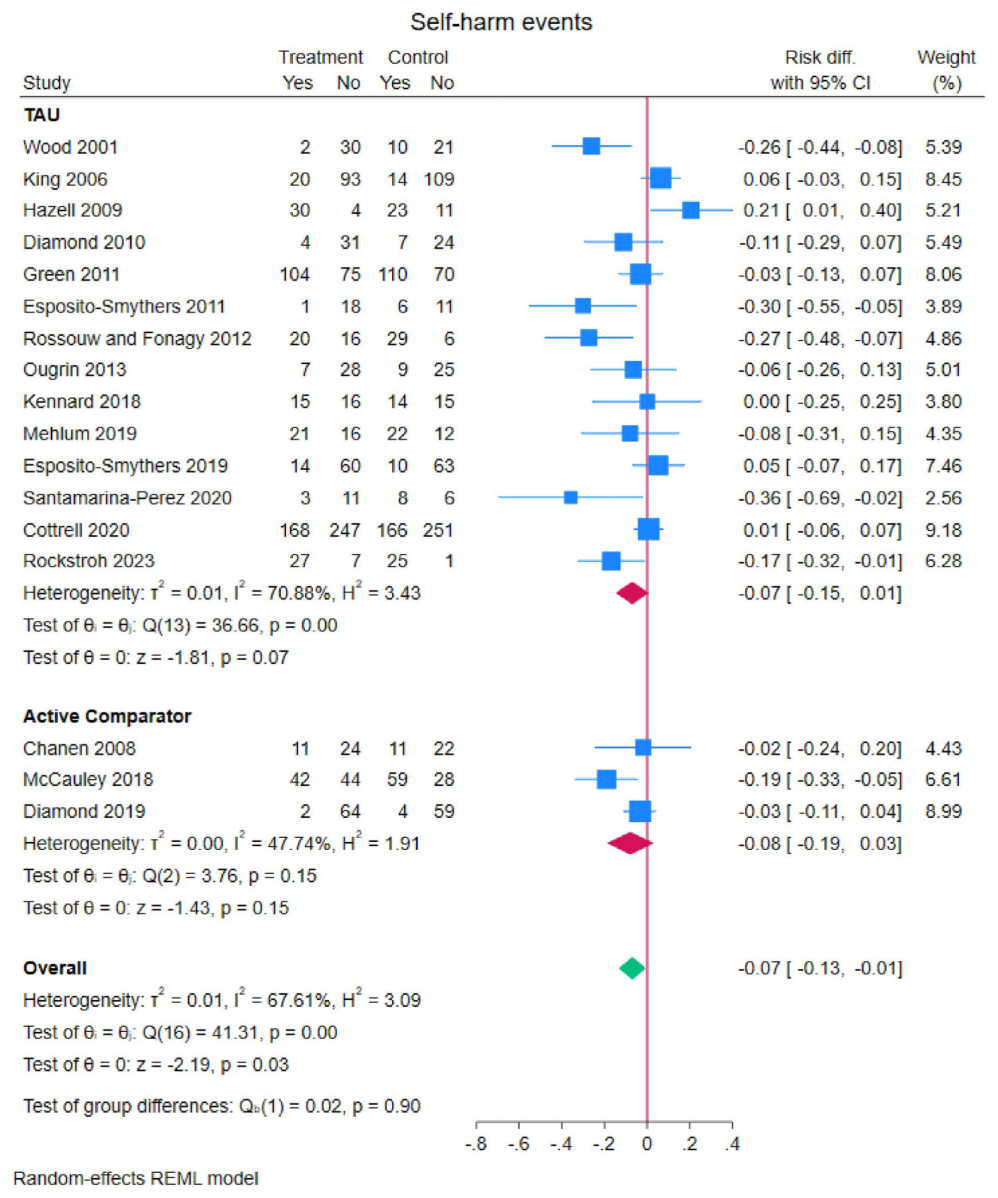

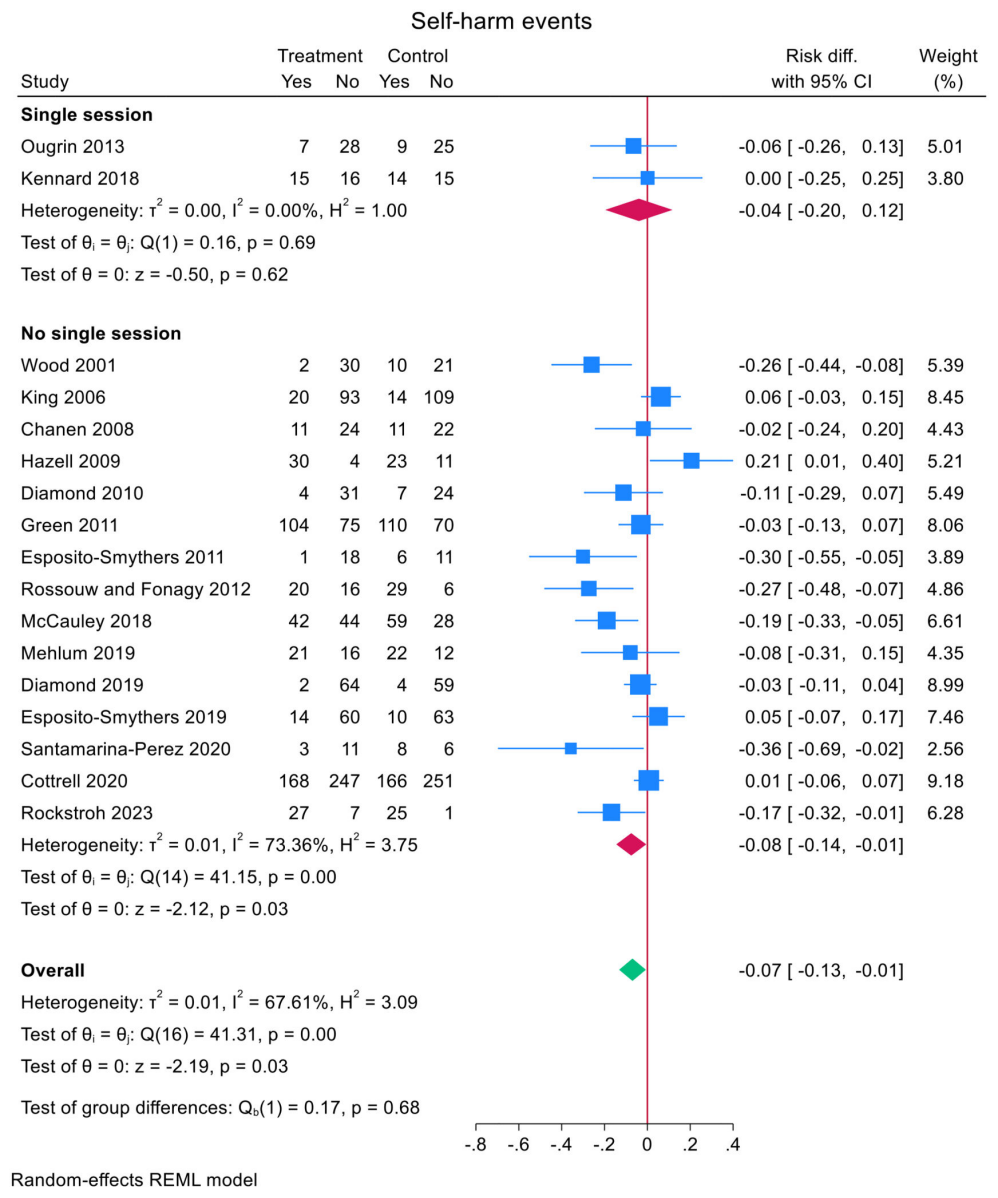
Effect of TIs versus comparator condition on repeat self-harm in adolescents.

Across 17 RCTs, 38.5% (491/1275) of adolescents who received a TI repeated self-harm compared to 41.8% (527/1261) of adolescents who did not (Figure 3). Heterogeneity between studies was moderate to high (*I*^2^ = 67.6%). The pooled risk difference for repeat self-harm in the TI group compared to comparator was −0.07 (95% CI, −0.132 to −0.007, *z* = −2.19, *p* = 0.028). Including total bias as a covariate in a meta-regression, showed a non-significant small effect (−0.021 (95% CI, −0.067 to 0.025), *z* = 0.88, *p* = 0.38).

A subgroup analysis showed that self-harm risk reduction was slightly larger in studies with active comparator (*N* = 3) (−0.079, (95% CI, −0.132 to −0.007), *z* = −1.43 *p* = 0.15) compared to studies with treatment as usual (*N* = 14) (−0.070 (95% CI, −0.146 to 0.006), *z* = −1.81, *p* = 0.07) (Figure 3). A meta-regression did not reveal significant differences between the two groups (mean difference 0.014 (95% CI, −0.176 to 0.149), *z* = 0.16, *p* = 0.87).

A subgroup analysis showed that self-harm risk reduction was slightly smaller in studies where the TI was a single session intervention (*N* = 2) (−0.040, (95% CI, −0.196 to 0.177), *z* = −0.50, *p* = 0.62) compared to studies that were not (*N* = 15) (−0.075 (95% CI, −0.145 to −0.006), *z* = −2.12, *p* = 0.034) (Figure 3). A meta-regression did not reveal significant differences between the two groups (mean difference −0.038 (95% CI, −0.263 to 0.187), *z* = −0.33, *p* = 0.74).

**Figure 4:**
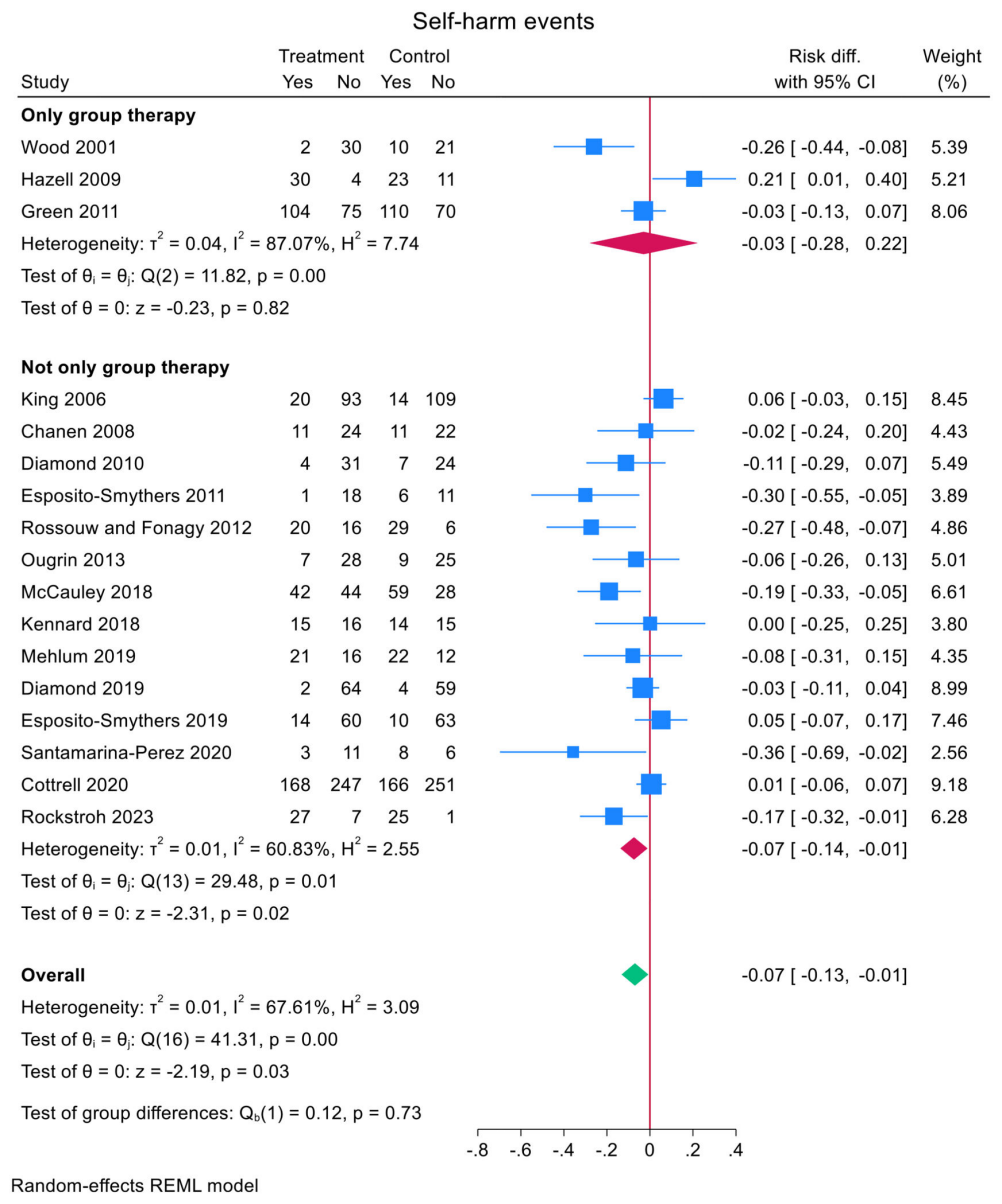
Effect of only group therapy versus not only group therapy on repeat self-harm.

A subgroup analysis showed that self-harm risk reduction was slightly smaller in studies with group therapy (*N* = 3) (−0.029, (95% CI, −0.281 to 0.223), *z* = −0.23, *p* = 0.82) compared to studies that were not group therapy (*N* = 14) (−0.074 (95% CI, −0.137 to −0.011), *z* = −2.31, *p* = 0.021) (Figure 4). A meta-regression did not reveal significant differences between the two groups (mean difference 0.050 (95% CI, −0.216 to 0.116), *z* = −0.59, *p* = 0.56).

**Figure 5:**
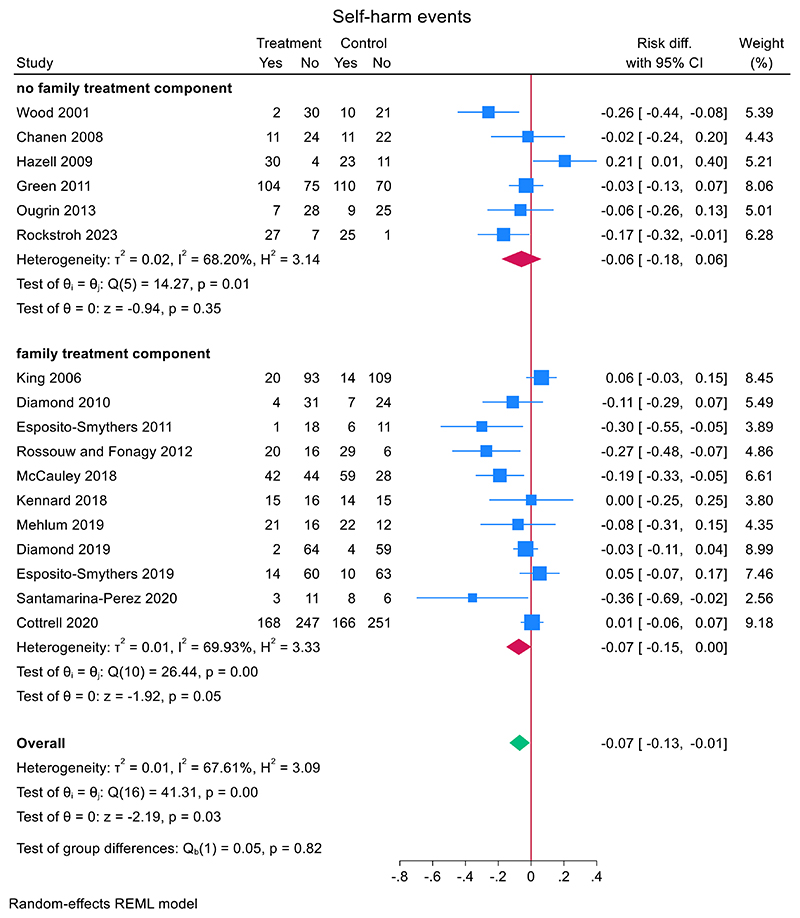
Effect of TIs with family treatment components versus TIs with no family treatment components on repeat self-harm.

There were little differences in risk reduction between trials with family treatment components (*N* = 11) mean risk difference: −0.075 (95% CI, −0.151 to 0.002, *z* = −1.92, *p* = 0.055) and no family treatment components (*N* = 6) mean risk difference: −0.058 (95% CI, −0.180 to 0.063, *z* = −0.94, *p* = 0.35) (Figure 5). A meta-regression revealed no significant differences between both groups (estimated mean difference between family components present and not present: −0.019 (95% CI, −0.156 to 0.118, *z* = −0.27, *p* = 0.79)).

### Suicide attempts meta-analysis

**Figure 6:**
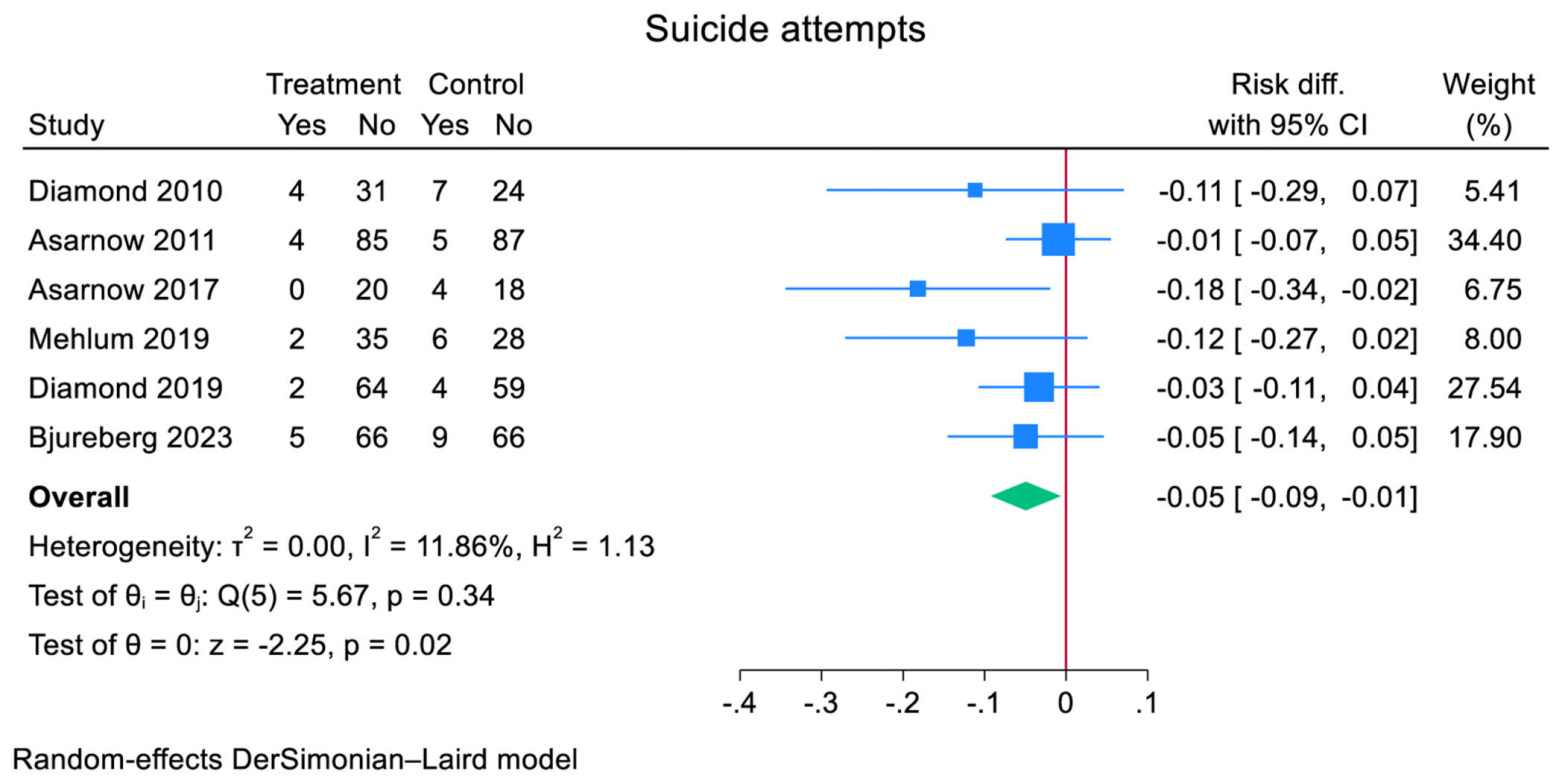
Effect of TI for self-harm versus comparator on individual suicide attempts in adolescents.

Across six RCTs with data specifically on individual suicide attempts, 17 adolescents (5.3%, 17/318) in the TI group attempted suicide compared to 35 (11.0%, 35/317) adolescents in the comparator group. Heterogeneity between studies was small (*I*^2^ = 29.4%). The pooled risk difference for suicide attempts post TI compared to a comparator was −0.046 (95% CI, −0.086 to −0.007, *z* = −2.29, *p* = 0.022). All RCTs were TIs of individual therapy with family treatment components (Figure 6).

### Publication bias and sensitivity analyses

**Figure 7:**
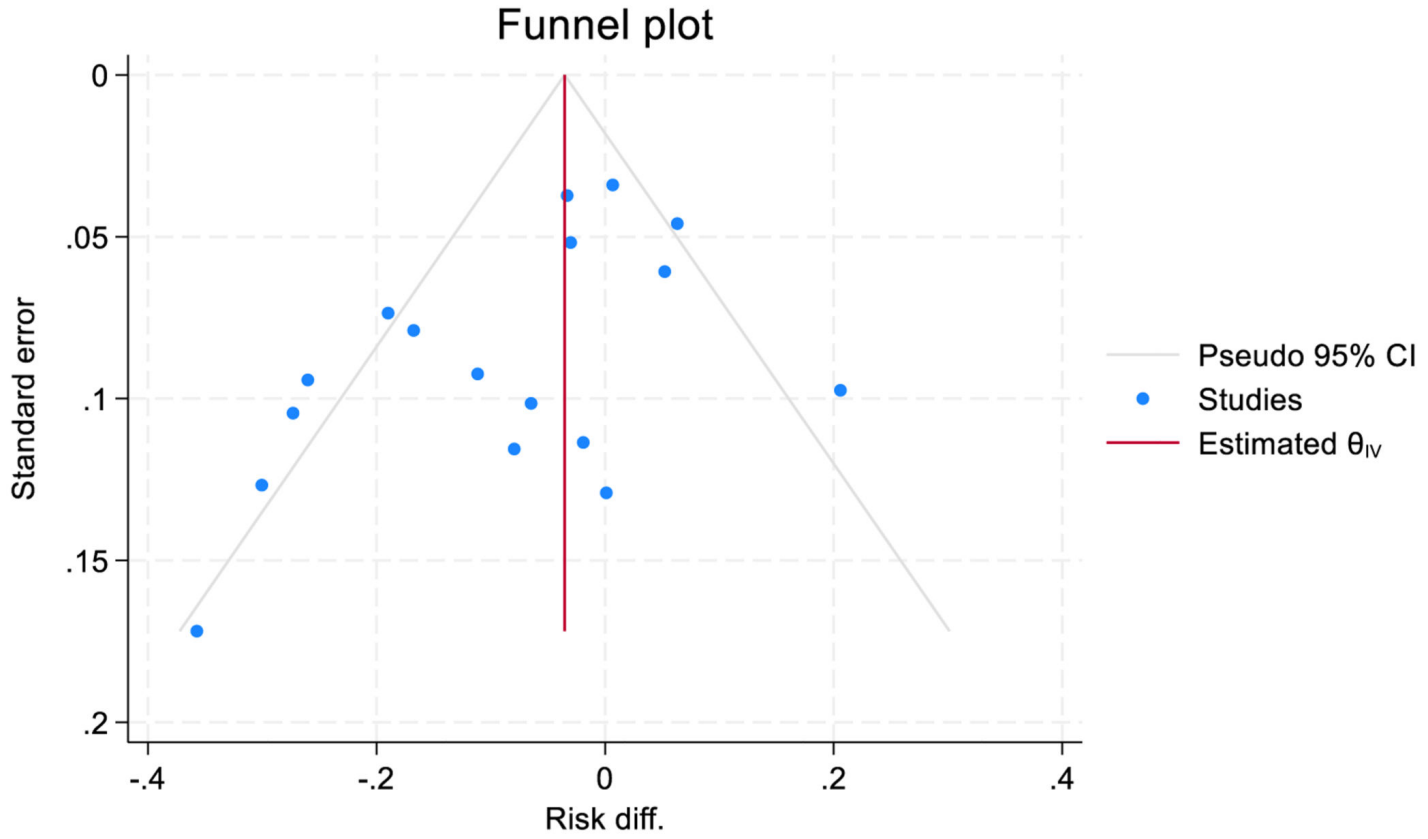
Funnel plot with pseudo-95% confidence limits for meta-analysis from RCTs with data on self-harm only.

A leave-one-out sensitivity analysis showed that individual studies do not strongly influence the overall estimate of self-harm, suicide attempts, or mortality. The change range was from −0.057 to −0.081 for self-harm, −0.006 to −0.004 for mortality and −0.037 to 0.067 for suicide attempts. Confidence intervals contain the overall effect estimate of each outcome based on all studies. A funnel plot suggests some possible small bias towards lower risk reductions (Figure 7), but a ‘trim and fill’ analysis did not estimate any missing study. Publication bias could only be assessed for self-harm studies.

## Discussion

This systematic review and meta-analysis of RCTs of TIs for adolescents after self-harm, to our knowledge, provides the first pooled risk difference for mortality in adolescents who received a TI compared to a comparator. We found that the risk difference of subsequent mortality was not significantly reduced after TI. This, however, needs to be considered in the context of a very low number of total participant deaths (*n* = 21), which limits the stability of estimates and requires these findings to be viewed as suggestive.

It is not clear, nor did we aim to understand why deaths may have occurred in RCTs, however, one reason may be that participant inclusion criteria led to adolescents recruited into trials who were more likely to die by suicide or other causes of death. In addition, the small number of deaths across RCTs may suggest that participation in a trial may have a protective component against death.

We identified a small but statistically significant important reduction in risk of both individual self-harm repetition and attempted suicide following TI. This identified reduction in risk of repeat self-harm after TI in adolescents aligns with recent meta-analyses (2015 and 2019) (Kothgassner et al., 2020; Ougrin et al., 2015), but our analysis included six new large RCTs and highlights stability and replication of treatment effect over time (Bjureberg et al., 2023; Diamond et al., 2019; Esposito-Smythers et al., 2019; Goldstein et al., 2024; Santamarina-Perez et al., 2020; Stallard et al., 2024).

Of importance is the identification that TIs led to reduced individual episodes of suicide attempts specifically and this has not been previously found. This may indicate similar treatment responses for both self-harm, and suicide attempts, suggesting that intervention strategies can successfully target both constructs, but this requires future replication with more adolescents.

### Implications for clinical practice and future research

It is crucial in future RCTs of TIs for adolescents after self-harm that mortality is an outcome captured and reported openly. Research needs to also aim to explore and understand why deaths may occur in participating adolescents.

Although significant differences for both self-harm and suicide attempts were small, these can translate into meaningful clinical outcomes for adolescents in clinical services. Any reduction in repeat self-harm and attempted suicide episodes is likely to have substantial effects on young people, their families, and health and care services, especially as engagement of young people with TIs has improved and digital delivery of psychological therapies is developing (Kennard et al., 2018; Yuan et al., 2019).

The recently updated National Institute for Health and Care Excellence guidance for self-harm recommends DBT-A is considered in adolescents with emotion dysregulation and frequent self-harm and this should translate into a benefit for adolescents because services are often informed by latest clinical guidance (National Institute for Health and Care Excellence, 2022). This mention of DBT-A in national guidance should facilitate the adoption of this particular therapeutic approach, but more trial evidence is needed with heterogenous participant samples and longer follow-up times (National Institute for Health and Care Excellence, 2022). Individual participant data meta-analyses would allow understanding of whether reductions in self-harm and suicide attempts occur in certain subgroups of adolescents compared to others after TI (Witt et al., 2021).

Our subgroup analyses found no significant differences for the risk of repeat self-harm in RCTs delivering group treatment compared to non-group; in RCTs with active comparators compared to treatment as usual; single-session TIs compared to non-single session; and in TIs with family treatment components compared to no family treatment components.

### Strengths and limitations

This review was conducted and reported adhering to PRISMA guidance for meta-analyses, and searches were updated to reduce the likelihood of missed trials. Study screening, selection, data extraction, and risk of bias scoring were conducted by two independent authors. Study authors were personally contacted to obtain mortality data because data were either not published at the time of searches or reported in the original trial paper. A leave-one-out analysis suggested that individual studies did not strongly influence the pooled estimate for mortality, repeat self-harm, and attempted suicide.

There are, however, several limitations to note. First, dependent on when trial recruitment closed there were different mortality follow-up time periods for RCTs which introduces potential bias. Analysis was combined across diverse intervention types and subpopulations of self-harming youth, and generally brief follow-up intervals were likely insufficient to detect effects on mortality. The timing of TI after self-harm varied across trials and in some trials not all adolescents included had self-harmed. In addition, further mortality analyses were restricted due to a small number of events across trials. There was diversity across TIs which led to a random effects model being used that assumed a distribution of treatment effect and thus the estimated effect sizes need to be considered as average treatment effects. Although not an aim of this review future meta-analyses should examine treatment effects within specific groups of TIs. Meta-regression for outcomes less than 10 studies per covariate need to be treated with care (Higgins et al., 2019). All RCTs were conducted in developed countries and these findings are likely thus not applicable to lower-and-middle income country clinical settings.

## Conclusion

In this systematic review and meta-analysis of 3470 randomised adolescents, therapeutic intervention did not lead to reduced risk of future death in adolescents after self-harm, but this needs to be interpreted with caution. There were significant effects identified for therapeutic interventions on both individual repeat self-harm and suicide attempts. Future trials should attempt to recruit heterogeneous samples and capture long-term outcome data.

## Supplementary Material

Additional supporting information can be found online in the Supporting Information section at the end of this article.

Supporting Information S1

## Figures and Tables

**Table 1:** Characteristics of included RCTs.

Study (country)	Age (years)	Intervention-type	Comparator arm	Trial setting	Family treatment component	No. participants randomized to the intervention	No. participants randomized to comparator
Asarnow et al. (2011) (USA)^a^	Mean: 14.7	Family intervention for suicide prevention	Enhanced TAU	Hospital setting with telephone follow-up	Yes	89	92
Asarnow et al. (2017) (USA)^a^	Mean: 14.6	CBT and DBT informed the family treatment	Enhanced TAU	Outpatient hospital setting	Yes	20	22
Bjureberg et al. (2023) (Sweden)^a^	Mean: 15.0	Online CBT informed emotion regulation programme	TAU	CAMHS	Yes	84	82
Chanen et al. (2008) (Australia)	15–18	Cognitive analytical therapy	Manualised good clinical care	Specialist young people’s mental health clinic	No	44	42
Cottrell et al. (2020) (UK)	11–17	Family therapy	TAU	CAMHS	Yes	416	416
Diamond et al. (2010) (USA)	12–17 (mean: 15.1)	Attachment based family therapy	Enhanced usual care	Children’s hospital	Yes	35	31
Diamond et al. (2019) (USA)^a^	12–18 years (mean: 14.9)	Attachment based family therapy	Family enhanced nondirective supportive therapy	Children’s hospital	Yes	66	63
Esposito-Smythers et al. (2011) (USA)^a^	13–17 (mean: 15.7)	Integrated-CBT	Enhanced TAU	Outpatient therapist-led sessions	Yes	20	20
Esposito-Smythers et al. (2019) (USA)^a^	12–18 (mean: 14.9)	Family focused-CBT	Enhanced TAU	Outpatient therapist led	Yes	74	73
Goldstein et al. (2024) (USA)^a^	Mean: 16.1	DBT for adolescents	Standard of care psychotherapy	Outpatient therapist led	No	47	53
Green et al. (2011) (UK)^a^	12–17	Developmental group psychotherapy	TAU	Therapist-led in CAMHS	No	183	183
Hazell et al. (2009) (Australia)^a^	12–16	Group therapy	TAU	CAMHS	No	35	37
Kennard et al. (2018) (USA)^a^	Mean: Treatment arm 14.9 versus 15.3	As safe as possible (ASAP) focussed on emotion regulation and safety planning and post-discharge mobile app	TAU	Inpatient therapist led	Yes	34	32
King et al. (2006) (USA)^a^	Mean: 15.3	Youth nominated support team for suicidal adolescents I	TAU	Hospital-based	Yes	151	138
King et al. (2019) (USA)^a^	13–17 (mean: 15.6)	Youth nominated support team for suicidal adolescents II	TAU	Hospital-based	Yes	223	225
McCauley et al. (2018) (USA)^a^	Mean: 14.9	DBT	Individual and group supported therapy	Medical centres	Yes	86	87
Mehlum et al. (2019) (Norway)^a^	Mean: 15.6	DBT-A	Enhanced TAU	CAMHS	Yes	39	38
Ougrin et al. (2013) (UK)	12–18	Therapeutic assessment	TAU	CAMHS	No	35	35
Pineda and Dadds (2013) (Australia)^a^	12–17	Family intervention: Resourceful adolescent parent programme	TAU	Specialist public mental health service	Yes	24	24
Rockstroh et al. (2023) (Germany)^a^	12–17 (mean: 14.9)	Cutting down programme—CBT and DBT informed	TAU	CAMHS	No	37	37
Rossouw & Fonagy (2012) (UK)	12–17 (mean: 14.7)	MBT-A	TAU	CAMHS	Yes	40	40
Santamarina-Perez et al. (2020) (Spain)^a^	Mean: Treatment arm 15.3 versus 15.2	DBT-A	Enhanced TAU	Community mental health clinic	Yes	18	17
Stallard et al. (2024) (UK)^ a^	Mean: 15.6	Co-designed smartphone app informed by CBT and DBT	TAU	CAMHS	No	85	85
Wood et al. (2001) (UK)	Mean: Treatment arm 14.2 versus 14.3	Developmental group therapy	TAU	CAMHS	No	32	31

Abbreviations: CAMHS, Child and Adolescent Mental Health Services; TAU, treatment as usual.

a Authors provided outcome data.

**Table 2:** Risk of bias scores for included randomised controlled trials (RCT).

Risk of bias domain	Asarnow et al. (2011)	Asarnow et al. (2017)	Bjureberg et al. (2023)	Chanen et al. (2008)	Cottrell et al. (2020)	Diamond et al. (2010)	Diamond et al. (2019)	Esposito-Smythers et al. (2011)	Esposito-Smythers et al. (2019)
Random sequence generation (selection bias)	Low	Low	Low	Low	Low	Low	Low	Low	Low
Allocation concealment (selection bias)	Low	Low	Low	Low	Low	Low	Low	Unclear	Low
Blinding of participants and researchers (performance bias)	Unclear	Unclear	Low	Low	Low	High	Unclear	Unclear	Unclear
Blinding of outcome assessment (detection bias)	Low	High	Low	Low	Low	High	Low	High	Unclear
Incomplete outcome data (attrition bias)	Low	Low	Unclear	Low	Low	High	Low	Low	Low
Selective reporting (reporting bias)	Low	Low	Unclear	Low	Low	Low	High	Low	Low
**Risk of bias domain**	**Goldstein et al. (2024)**	**Green et al. (2011)**	**Hazell et al. (2009)**	**Kennard et al. (2018)**	**King et al. (2006)**	**King et al. (2019)**	**McCauley et al. (2018)**	**Mehlum et al. (2019**	**Ougrin et al. (2013)**
Random sequence generation (selection bias)	Low	Low	Unclear	Low	Low	Low	Low	Low	Low
Allocation concealment (selection bias)	Unclear	Low	Unclear	Unclear	Unclear	Unclear	Low	Unclear	Unclear
Blinding of participants and researchers (performance bias)	High	Low	High	Unclear	Unclear	Unclear	High	Unclear	Low
Blinding of outcome assessment (detection bias)	Low	Low	Low	Low	High	Low	Low	Low	Low
Incomplete outcome data (attrition bias)	Low	Low	Unclear	Low	Unclear	Low	Low	Low	Low
Selective reporting (reporting bias)	Unclear	Low	Low	Low	Low	High	Low	High	Low
**Risk of bias domain**		**Pineda and Dadds (2013)**	**Rockstroh et al. (2023)**		**Rossouw & Fonagy (2012)**		**Santamarina-Perez et al. (2020)**	**Stallard et al. (2024)**	**Wood et al. (2001)**
Random sequence generation (selection Low bias)			Low		Low	Low	Low		Low
Allocation concealment (selection bias) Low			Low		Low	Low	Low		Unclear
Blinding of participants and researchers Unclear (performance bias)			High		Low	High	Low		Unclear
Blinding of outcome assessment (detection bias)		Low	Low		Low	Low	Unclear		Low
Incomplete outcome data (attrition bias)		Low	High		Low	Low	Low		Unclear
Selective reporting (reporting bias)		Low	Low		Low	Low	Low		Low

## Data Availability

The corresponding author will consider appropriate data sharing requests.

## Key points

- Therapeutic intervention (TI) after self-harm in adolescents did not impact on future mortality
- For the first time we found TI led to reduced episodes of suicide attempts
- We confirmed that TIs lead to reduced individual repetition of self-harm in adolescents
- These findings, in particular, for repeat self-harm and suicide attempts, highlight the need for enhanced provision of TIs in mental health services
