# Supplementary material for: Mortality in adolescents after therapeutic intervention for self‐harm: A systematic review and meta‐analysis
Source: JCPP Adv. 2025 Jan 23;5(3):e12302. doi: 10.1002/jcv2.12302 (PMC7617579; doi:10.1002/jcv2.12302)
Supplement: Supplementary file 1 — Supporting Information S1 [file JCV2-5-e12302-s001.docx]

**Supporting Information**

**Appendix S1.** Electronic database search strategies.

**Database: Ovid MEDLINE(R) ALL <1946 to June 19, 2024>**
**Search Strategy:**
**1**  exp Self-Injurious Behavior/ (86748)
**2**  exp Poisoning/ (171697)
**3**  drug overdose/ (15141)
**4**  self harm*.ti,ab,kf. (9777)
**5**  self injur*.ti,ab,kf. (7158)
**6**  self mutilat*.ti,ab,kf. (1966)
**7**  head bang*.ti,ab,kf. (177)
**8**  headbang*.ti,ab,kf. (40)
**9**  self destruct*.ti,ab,kf. (2284)
**10**  overdos*.ti,ab,kf. (31724)
**11**  self inflict*.ti,ab,kf. (3018)
**12**  self poison*.ti,ab,kf. (2120)
**13**  parasuicid*.ti,ab,kf. (660)
**14**  ((self or themself or themselv*) adj3 cut*).ti,ab,kf. (1023)
**15**  or/1-14 (295144)
**16**  exp Adolescent/ (2254691)
**17**  exp Child/ (2210751)
**18**  youth*.ti,ab,kf. (112404)
**19**  teenage*.ti,ab,kf. (25943)
**20**  adolescent*.ti,ab,kf. (348687)
**21**  child*.ti,ab,kf. (1753259)
**22**  juvenile*.ti,ab,kf. (99506)
**23**  teen*.ti,ab,kf. (37340)
**24**  or/16-23 (4095825)
**25**  exp Therapeutics/ (5345441)
**26**  exp Crisis Intervention/ (6334)
**27**  therap*.ti,ab,kf. (3813364)
**28**  interven*.ti,ab,kf. (1494409)
**29**  treatment*.ti,ab,kf. (5789554)
**30**  or/25-29 (11672451)
**31**  15 and 24 and 30 (26893)
**32**  exp Randomized Controlled Trial/ (617154)
**33**  randomi#e.ti,ab,kf. (2249)
**34**  controlled clinical trial.pt. (95556)
**35**  randomized.ab. (649702)
**36**  placebo.ab. (249258)
**37**  randomly.ab. (435841)
**38**  trial.ab. (702510)
**39**  or/32-38 (1690705)
**40**  31 and 39 (2153)
**41**  limit 40 to dt=20230131-20240620 [January 31st, 2023 to June 20th, 2024] (116)

**Database: APA PsycInfo <1806 to June Week 2 2024>**
**Search Strategy:**
**1**  exp Self-Injurious Behavior/ (8197)
**2**  exp Self-Mutilation/ (8197)
**3**  exp Drug Overdoses/ (3175)
**4**  exp suicidal ideation/ or exp suicide/ or exp suicide, attempted/ (48535)
**5**  self harm*.ti,ab. (7871)
**6**  self poison*.ti,ab. (679)
**7**  headbang*.ti,ab. (36)
**8**  head bang*.ti,ab. (178)
**9**  self injur*.ti,ab. (8050)
**10**  self destruct*.ti,ab. (3191)
**11**  overdos*.ti,ab. (7256)
**12**  self mutilat*.ti,ab. (1398)
**13**  self inflict*.ti,ab. (893)
**14**  parasuicid*.ti,ab. (741)
**15**  ((self or themself or themselv*) adj3 cut*).ti,ab. (568)
**16**  or/1-15 (70708)
**17**  exp Adolescent Psychiatry/ or exp Adolescent Psychotherapy/ or exp Adolescent Psychology/ or exp Adolescent Psychopathology/ (22627)
**18**  exp Child Psychology/ or exp Child Psychopathology/ or exp Child Psychiatry/ or exp Child Psychotherapy/ (26003)
**19**  adolescent*.ti,ab. (255502)
**20**  child*.ti,ab. (798895)
**21**  youth*.ti,ab. (125096)
**22**  teenage*.ti,ab. (14999)
**23**  teen*.ti,ab. (25150)
**24**  juvenile*.ti,ab. (28134)
**25**  or/17-24 (1029180)
**26**  exp Treatment/ (1369579)
**27**  exp Crisis Intervention/ or exp Intervention/ (149153)
**28**  therap*.ti,ab. (450571)
**29**  interven*.ti,ab. (515048)
**30**  treatment*.ti,ab. (719761)
**31**  or/26-30 (1885687)
**32**  double-blind.ti,ab. (25556)
**33**  random* assigned.ti,ab. (43606)
**34**  control*.ti,ab. (812486)
**35**  randomly.ab. (87063)
**36**  placebo.ab. (44240)
**37**  trial.ab. (119078)
**38**  controlled clinical trial.ab. (1416)
**39**  16 and 25 and 31 (9990)
**40**  or/32-38 (935950)
**41**  39 and 40 (1845)
**42**  limit 41 to yr="2023 -Current" (159)

**Database: Embase Classic+Embase <1947 to 2024 June 19>**
**Search Strategy:**
**1**  exp automutilation/ (27084)
**2**  exp intoxication/ (513900)
**3**  exp drug overdose/ (37655)
**4**  exp suicide/ or exp suicidal ideation/ or exp suicide attempt/ (128184)
**5**  self harm*.ti,ab,kw. (12717)
**6**  headbang*.ti,ab,kw. (59)
**7**  head bang*.ti,ab,kw. (267)
**8**  self injur*.ti,ab,kw. (9040)
**9**  self destruct*.ti,ab,kw. (2938)
**10**  overdos*.ti,ab,kw. (47837)
**11**  self mutilat*.ti,ab,kw. (2605)
**12**  self inflict*.ti,ab,kw. (3848)
**13**  self poison*.ti,ab,kw. (2836)
**14**  parasuicid*.ti,ab,kw. (926)
**15**  ((self or themself or themselv*) adj3 cut*).ti,ab,kw. (1333)
**16**  or/1-15 (667428)
**17**  exp adolescent/ (1992514)
**18**  exp child/ (3629726)
**19**  youth*.ti,ab,kw. (135107)
**20**  teenage*.ti,ab,kw. (37054)
**21**  teen*.ti,ab,kw. (53163)
**22**  adolescent*.ti,ab,kw. (463408)
**23**  child*.ti,ab,kw. (2389551)
**24**  juvenile*.ti,ab,kw. (129549)
**25**  or/17-24 (5280068)
**26**  exp therapy/ (11399162)
**27**  exp crisis intervention/ (7319)
**28**  exp intervention study/ (68303)
**29**  interven*.ti,ab,kw. (2065843)
**30**  treatment*.ti,ab,kw. (8386926)
**31**  therap*.ti,ab,kw. (5451698)
**32**  or/26-31 (17677480)
**33**  16 and 25 and 32 (59798)
**34**  exp randomized controlled trial/ (832638)
**35**  randomi#e.ti,ab,kw. (3315)
**36**  randomized.ab. (945718)
**37**  placebo.ab. (373653)
**38**  randomly.ab. (583132)
**39**  trial.ab. (1045530)
**40**  controlled clinical trial.ab. (18522)
**41**  or/34-40 (2318138)
**42**  33 and 41 (4555)
**43**  limit 42 to embase (3158)
**44**  limit 43 to dd=20230130-20240620 [January 30th, 2023 to June 20th, 2024] (14)

Cochrane search strategy

#1 ("self harm"):ti,ab,kw 902

#2 ("self mutilation"):ti,ab,kw 63

#3 ("self injur*"):ti,ab,kw 0

#4 (suicid*):ti,ab,kw 9089

#5 ("self poison"):ti,ab,kw 2

#6 (headbang*):ti,ab,kw 5

#7 (head bang*):ti,ab,kw 100

#8 (self destruct*):ti,ab,kw 301

#9 (overdos*):ti,ab,kw 2423

#10 (parasuicid*):ti,ab,kw 87

#11 ("self cutting"):ti,ab,kw 12

#12 (nonsuicidal):ti,ab,kw 293

#13 ("non suicidal"):ti,ab,kw 205

#14 Self-Injurious Behavior 810

#15 Self Mutilation 180

#16 #1 or #2 or #3 or #4 or #5 or #6 or #7 or #8 or #9 or #10 or #11 or #12 or #13 or #14 or #15 12432

#17 (Adolescent*):ti,ab,kw 173030

#18 (Child*):ti,ab,kw 210893

#19 (Youth*):ti,ab,kw 11071

#20 (Teenage*):ti,ab,kw 1815

#21 (Teen*):ti,ab,kw 3687

#22 (Juvenile*):ti,ab,kw 5193

#23 Adolescent 167963

#24 Child 216487

#25 #17 or #18 or #19 or #20 or #21 or #22 or #23 or #24 344263

#26 (Treatment*):ti,ab,kw 1013903

#27 (Therap*):ti,ab,kw 998562

#28 (Interven*):ti,ab,kw 644785

#29 Therapeutics 27978

#30 Crisis Intervention 2838

#31 #26 or #27 or #28 or #29 or #30 1610058

#32 #16 AND #25 AND #31 with Cochrane Library publication date Between Jan 2023 and Jun 2024
